# Metabolism-Based Herbicide Resistance to Mesosulfuron-methyl and Identification of Candidate Genes in *Bromus japonicus*

**DOI:** 10.3390/plants13131751

**Published:** 2024-06-25

**Authors:** Qi Li, Hengzhi Wang, Jinping Yu, Wei Zhang, Wenlei Guo, Yixue Liu

**Affiliations:** 1 Institute of Plant Protection, Tianjin Academy of Agricultural Sciences, Tianjin 300381, China; liqi0309@hotmail.com (Q.L.); yujp_love@163.com (J.Y.); weiyzhang001@126.com (W.Z.); 2 Key Laboratory of Pesticide Toxicology and Application Technique, College of Plant Protection, Shandong Agricultural University, Tai’an 271018, China; wanghz@sdau.edu.cn; 3 Guangdong Provincial Key Laboratory of High Technology for Plant Protection, Plant Protection Research Institute, Guangdong Academy of Agricultural Sciences, Guangzhou 510640, China

**Keywords:** abiotic stress, *Bromus japonicus*, mesosulfuron-methyl, metabolic resistance, transcriptomics, molecular mechanisms

## Abstract

The evolved resistance of *Bromus japonicus* Houtt. to ALS-inhibiting herbicides is well established. Previous studies have primarily focused on target-site resistance; however, non-target-site resistance has not been well characterized. This investigation demonstrated that *ALS* gene sequencing did not detect any previously known resistance mutations in a mesosulfuron-methyl-resistant (MR) population, and notably, treatment with the P450 monooxygenase (P450) inhibitor malathion markedly heightened susceptibility to mesosulfuron-methyl. Utilizing UPLC-MS/MS analysis confirmed elevated mesosulfuron-methyl metabolism in MR plants. The integration of Isoform Sequencing (Iso-Seq) and RNA Sequencing (RNA-Seq) facilitated the identification of candidate genes associated with non-target sites in a subpopulation with two generations of herbicide selection. Through qRT-PCR analysis, 21 differentially expressed genes were characterized, and among these, 10 genes (comprising three P450s, two glutathione *S*-transferases, one glycosyltransferase, two ATP-binding cassette transporters, one oxidase, and one hydrolase) exhibited constitutive upregulation in resistant plants. Our findings substantiated that increased herbicide metabolism is a driving force behind mesosulfuron-methyl resistance in this *B. japonicus* population.

## 1. Introduction

Weeds are a serious threat to food security, as they reduce crop yields worldwide [1]. Because of their high efficiency, simplicity, and low cost, herbicides have become the most important means of weed control in modern agriculture. However, their long-term and widespread use has resulted in weed resistance to herbicides, which has become a critical global problem [2]. A better understanding of weed resistance mechanisms is crucial for monitoring resistance and weed management.

Weed resistance mechanisms can be divided into two main categories: target-site resistance (TSR) and non-target-site resistance (NTSR) [3,4]. TSR mainly results from mutations in genes encoding herbicide-binding sites, whereas NTSR is a form of resistance mechanism that reduces the amount of herbicide reaching the target site, such as decreased herbicide penetration into the plant, decreased rates of herbicide translocation, and increased rates of herbicide sequestration/metabolism [3,4,5,6,7,8]. Enhanced herbicide metabolism, also named metabolic resistance, has been reported in some important weed species [9], involving P450 monooxygenase (P450), glucosyltransferases (GT), glutathione S-transferases (GST), ATP-binding cassette (ABC) transporter, and peroxidase (POD) [6,7]. For example, enhanced metabolism by cytochrome P450s is likely a mechanism of resistance to pyroxsulam in *Bromus sterilis* [10]. Owing to these unpredictable processes, herbicide metabolic genes may have pleiotropic effects on weeds [11]. It has been reported that P450 is associated with fitness costs, although this link is sometimes nonexistent [12,13]. Metabolic resistance has the potential to endow plants with tolerance to a range of herbicides, irrespective of their mode of action, thus posing a significant risk to herbicide efficacy and sustainability [14]. Therefore, research on NTSR mechanisms is critical for continuous weed control.

Transcriptome sequencing (RNA-Seq) technology offers a robust approach for exploring the genetic mechanisms underlying herbicide stress responses in weeds [15,16], especially for identifying the genetic differences between herbicide-resistant and susceptible plants [17,18,19]. Recently, RNA-Seq has been used to identify several genes related to NTSR in weeds, such as *Lolium rigidum* Gaudin [17,20,21], *Alopecurus myosuroides* Huds. [22], *Beckmannia syzigachne* Steud. [23], *Descurainia sophia* L. [24], *Alopecurus aequalis* Sobol. [25], and *Myosoton aquaticum* L. [26]. Nevertheless, the limitations present in transcript reconstruction create significant challenges in computation and complicate the detection of splice events [27]. Recently, full-length transcriptomes that offer long reads extending up to 10 kb have been generated. This enables the precise reconstruction of full-length splice variants, resulting in a more reliable isoform dataset compared to that from RNA-Seq [28].

*Bromus japonicus* Houtt. is an annual grass weed that severely invades winter wheat fields in China [29]. Seedlings typically emerge during September and October, initiate flower in early May, and commence seed dispersal by early October [30]. A *B. japonicus* plant can produce approximately 1885 seeds, which are generally dispersed by wind and water flow because of their light weight. These seeds can germinate over a wide temperature range of 5 to 30 °C, under varying pH levels, and even in the absence of light [29]. *B. japonicus* competes with wheat, leading to at least a 30% yield loss in fields heavily infested with 640 plants m^−2^ [31]. Furthermore, extensive genetic variability has occurred among and within the Chinese populations of *B. japonicus*, which is likely to contribute significantly to their adaptability and infestation as a weed species [32]. Herbicides, as strong selective factors, can result in selection for resistance among populations in a short time, making resistant plants dominant. Currently, postemergence treatment with herbicides, such as flucarbazone-sodium, pyroxsulam, and mesosulfuron-methyl, have been widely adopted for controlling *B. japonicus*. Unfortunately, *B. japonicus* has developed a remarkable degree of target-site resistance to these herbicides in certain regions of China because of their extensive and persistent use [33,34]. In this study, we identified a population of *B. japonicus* (TJ07) that showed high resistance to the herbicide methyl 2-[(4,6-dimethoxypyrimidin-2-yl)carbamoylsulfamoyl]-4-(methanesulfonamidomethyl)benzoate (mesosulfuron-methyl), with no known ALS resistance mutations in surviving individuals. In addition, P450s may play an important role because the P450 inhibitor malathion can significantly reduce weed resistance to mesosulfuron-methyl. However, the genes responsible for metabolic resistance remain unclear. Therefore, we performed ultra-performance liquid chromatography-tandem mass spectrometry (UPLC-MS/MS) assays and second- and third-generation sequencing in this study to confirm metabolism-based resistance in TJ07 and to provide abundant genetic information on crucial herbicide-metabolizing enzymes.

## 2. Results

### 2.1. Mesosulfuron-methyl Dose–Response in the Absence and Presence of Malathion

**Figure 1:**
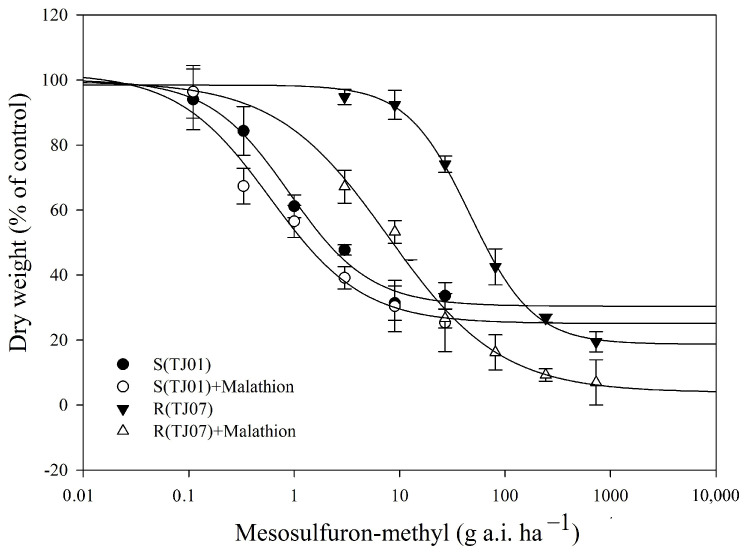
Dose–response curves depicting the dry weight of *B. japonicus* populations, MR (TJ07) and S (TJ01), were generated following treatment with various mesosulfuron-methyl dosages, both with (+) and without the addition of 1000 g a.i. ha^−1^ of malathion. The presented values are normalized to represent the percentage relative to the untreated control. Each data point represents the mean ± SE derived from two replicated experiments. The lines were fitted based on these mean values for clarity and accuracy by “four-parameter log-logistic equation”.

Whole-plant dose–response experiments demonstrated that the MR population had a significant resistance level (resistance index, RI = 51.8-fold) to mesosulfuron-methyl (Table 1). When malathion was applied alone (1000 g a.i. ha^−1^), there was no visual effect on MR or S seedling growth. However, malathion significantly reduced the resistance of the MR population to mesosulfuron-methyl. Under combined mesosulfuron-methyl and malathion treatments, the GR_50_ values of mesosulfuron-methyl decreased by 83.2% and 34% in the MR and S populations, respectively (Table 1, Figure 1). Malathion is typically used as a P450 indicator of herbicide metabolic resistance. These results indicated that P450s likely mediate enhanced mesosulfuron-methyl metabolism in the MR population.

### 2.2. ALS Gene Sequencing

ALS gene sequencing revealed no known ALS resistance mutations in MR plants (Table 2). Therefore, whole-plant resistance in the MR population may not be due to a TSR mechanism.

### 2.3. Mesosulfuron-methyl Absorption and Metabolism in B. japonicus Plants

**Figure 2:**
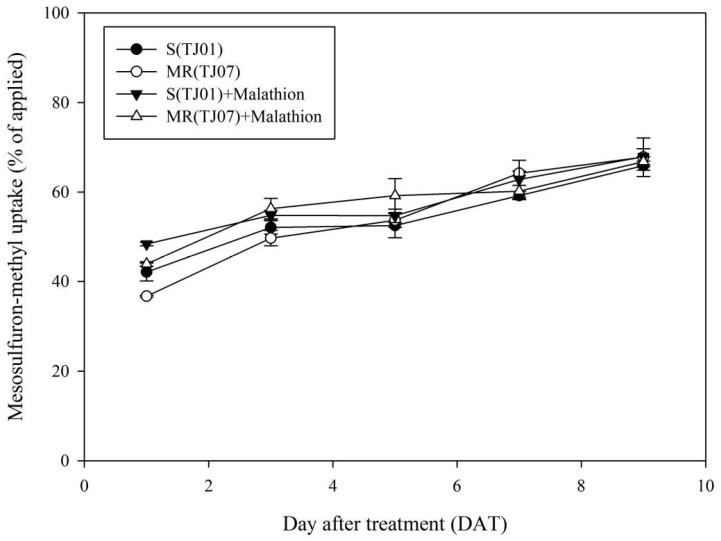
Mesosulfuron-methyl absorption rate in S (susceptible) and MR (resistant) *B. japonicus* at 1, 3, 5, 7, and 9 DAT. The absorption rate was calculated by dividing the absorption amount of mesosulfuron-methyl by the application amount of mesosulfuron-methyl multiplied by 100%.

UPLC-MS/MS analysis revealed no noteworthy disparity in the absorption rates of mesosulfuron-methyl between the MR and S populations in the absence of malathion pretreatment at 1, 3, 5, 7, and 9 days (Figure 2). Compared to the sole use of mesosulfuron-methyl, pre-treatment with malathion did not significantly enhance the absorption rate of mesosulfuron-methyl in either the MR or S populations (Figure 2). No statistically significant differences were observed between the MR and S populations at the same time points or under identical treatment conditions (Figure 2).

**Figure 3:**
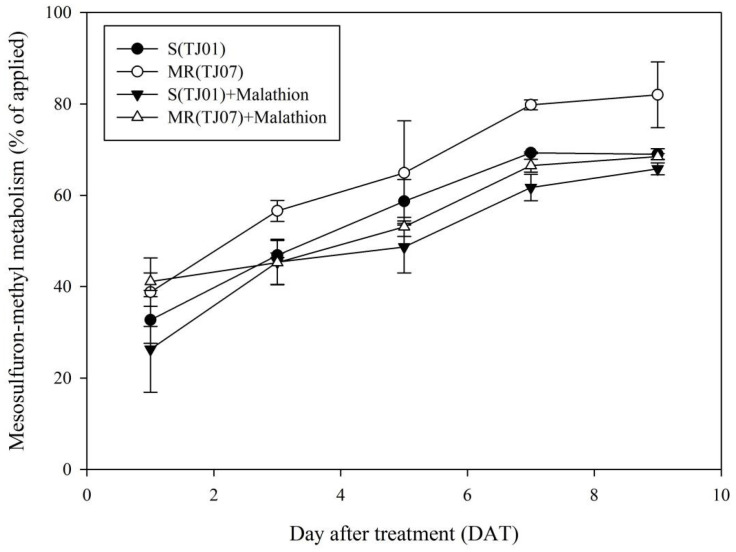
The metabolism rate of mesosulfuron-methyl in both susceptible (S) and resistant (MR) populations of *B. japonicus* at 1, 3, 5, 7, and 9 DAT was determined by dividing the metabolized quantity of mesosulfuron-methyl by the absorbed amount, and then multiplying by 100%.

Concerning herbicide metabolism, the mesosulfuron-methyl metabolism rate was significantly higher in the MR population without malathion pre-treatment compared to the S plants (Figure 3). Specifically, the metabolic rates in the MR plants at 3, 5, 7, and 9 DAT were 56.6%, 64.9%, 79.8%, and 82.0%, respectively, markedly surpassing those observed in the S plants, which were 46.9%, 58.7%, 69.3%, and 69.0%, respectively (Figure 3).

The application of malathion effectively minimized the disparity in metabolic rates between the MR and S populations. After malathion pre-treatment, the metabolic rate of MR plants decreased by 11.3, 11.8, 13.3, and 13.5% on the 3rd, 5th, 7th, and 9th day, respectively. However, the metabolic rate of S plants did not change significantly. Additionally, there was no notable disparity in the rate of mesosulfuron-methyl metabolism between the MR and S plants on the 3rd, 5th, 7th, and 9th day following malathion pre-treatment.

### 2.4. Transcriptome Sequencing, Assembly, and Functional Annotation

We employed Illumina sequencing technology in conjunction with PacBio SMRT sequencing to conduct a comprehensive analysis of gene expression disparities between MR and S populations under various treatments based on transcriptomic data. After quality filtering, approximately 249,000,000 high-quality clean data points were generated, ranging from 20,619,829 to 21,379,828 points per sample. The average Q30 percentage for each library was 94.43. After quality control, 413,904 circular consensus numbers with an average length of 1578 bp were obtained from PacBio SMRT sequencing. By eliminating polyA and chimeric sequences from the full-length sequences, we obtained 73,729 high-quality transcripts through clustering and error correction processes.

Based on redundant transcript sequences, 34,794 ORFs were predicted. Three different software programs (i.e., the coding potential calculator, coding non-coding index, and coding potential assessment tool) were used to predict the coding sequences. After excluding RNA molecules that possessed coding potential, a total of 2149 overlapping lncRNA predictions were pinpointed. Altogether, 33,220 assembled genes were annotated across eight databases, with the NR database yielding the highest degree of similarity (Table 3). Based on the NR database annotations, the assembled genes of *B. japonicus* were most similar to those of *Aegilops tauschii*, *Hordeum vulgare*, *Triticum urartu*, *Triticum aestivum*, *Brachypodium distachyon*, and *Oryza sativa* (Figure S1).

In the GO analysis, genes assigned to “biological process”, “cellular component”, and “molecular function” contained 50 functional subgroups (Figure S2). Among the 21 subgroups of biological processes, metabolic (53.38%) and cellular (50.73%) processes were the two major contributors. Cells (66.86%), cell parts (66.74%), and organelles (55.36%) were the three major contributors among the 15 subgroups of cellular components. Among the 14 molecular function subgroups, the catalytic activity (51.67%) and binding (51.57%) subgroups represented the two major contributors. KEGG analysis was used to annotate the information of the identified genes at the pathway level to further understand the function of the genes. They were assigned to 50 KEGG pathways, primarily involved in “Metabolism” (2120 genes, 73.66%), “Genetic information processing” (521 genes, 18.10%), “Cellular processes” (135 genes, 4.69%), “Environmental information processing” (54 genes, 1.88%), and “Organismal systems” (48 genes, 1.67%) (Figure S3).

### 2.5. Identification and Functional Analysis of DEGs

Transcript levels were reported as fragments per kilobase of transcript per million fragments mapped (RPKM). The full-length non-chimeric transcripts’ isoforms served as the reference sequence. Subsequently, we aligned the second-generation high-throughput sequencing data to this reference using RSEM software. Among the reads, 88.10% to 88.75% aligned to the reference sequence, while 49.77% to 52.04% mapped to multiple positions.

**Figure 4:**
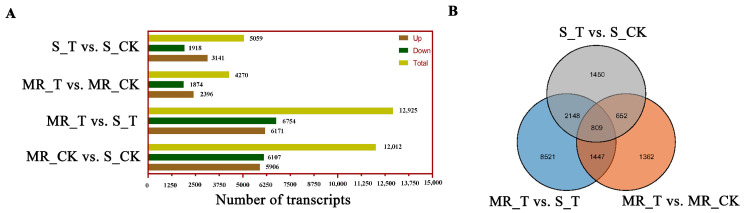
Statistics of the DEGs between the *B. japonicus* treatment groups. (**A**) The number of DEGs between the different groups. (**B**) Venn diagram showing the number of DEGs between MR (TJ07) and S (TJ01) samples in the three treatment comparisons.

We identified 4270 DEGs between the untreated and herbicide-treated MR samples with 2396 upregulated genes and 1874 downregulated genes. Compared with untreated S samples, 3141 upregulated and 1918 downregulated genes were found in herbicide-treated S samples. Compared with herbicide-treated samples, 6171 upregulated and 6754 downregulated genes were found in MR and S samples, respectively. Additionally, 5906 genes were upregulated, while 6107 were downregulated in untreated MR samples compared to untreated S samples (Figure 4A). Differential expression between MR and S samples in the three treatments (S_T vs. S_CK, MR_T vs. MR_CK, and MR_T vs. S_T) was evident in 809 contigs (Figure 4B).

Next, GO and KEGG enrichment analyses were performed on the annotated DEGs between the MR_T and S_T samples (Figure S4). In GO analysis, 9808 DEGs were enriched in the GO subgroups “metabolic process” (5467, 55.74%) and “cellular process” (5084, 51.83%) in biological processes; “cell” (6542, 66.70%), “cell part” (6528, 66.56%), and “organelle” (5394, 55.00%) in cellular components; and “catalytic activity” (5232, 53.34%) and “binding” (5123, 52.23%) in molecular function. Moreover, DEGs were enriched in 123 KEGG pathways. Among the top 15 enriched pathways of upregulated genes (Table 4), 34 upregulated genes were enriched in “Glutathione metabolism” pathways. Overall, annotation and enrichment analyses suggested that GST genes and other genes involved in metabolic/signaling pathways are vital for the metabolic resistance of *B. japonicus* against mesosulfuron-methyl.

### 2.6. Selection of Candidate Metabolic-Resistance Genes and Their Relative Expressions

We analyzed the constitutive and herbicide-inducible DEGs related to metabolic resistance in MR and S plants to identify and validate candidate genes. Based on metabolism-related functional annotations, we identified genes associated with detoxification (e.g., those encoding P450, GST, GT, ABC transporter, oxidase, hydrolase, and POD), resulting in the identification of 21 contigs (Table 5). Their expression levels were verified through two rounds of qRT-PCR.

Findings revealed that in the transcriptome sample group, 14 out of 21 candidate genes exhibited significantly higher expression levels in MR (TJ07) plants compared to S (TJ01) plants. In the parallel sample group, 11 out of 21 candidate genes were notably upregulated, with 10 contigs consistently upregulated in both groups (Table 5). They encoded proteins with homology to three P450s (*CYP71C4*, *CYP90D2*, and *CYP72A15*), two GSTs (*MEE6.28* and *GSTZ5*), one GT (*SGT31*), two ABC transporters (*ABCC10* and *ABCC2*), one oxidase, and one hydrolase.

## 3. Discussion

Mesosulfuron-methyl is a highly effective ALS-inhibiting herbicide extensively used in China over the last decade. However, extensive and persistent use has inevitably resulted in the development of herbicide resistance. Resistance to mesosulfuron-methyl has been identified in many monocotyledonous weeds such as *A. aequalis*, *B. japonicus*, *A. tauschii*, *Lolium multiflorum*, *Alopecurus japonicus*, and *B. syzigachne* [25,33,34,35,36,37,38]. With increasing levels of mesosulfuron-methyl resistance in weeds, there is an urgent need to elucidate potential resistance mechanisms. NTSR plays a crucial role in resistance to ACCase- and ALS-inhibiting herbicides [7,39].

In recent years, RNA-Seq technology has been instrumental in illuminating the mechanisms underlying metabolic herbicide resistance in weeds [24,25,26,40]. Unfortunately, the limitations of RNA-Seq in sequencing fragments of less than 300bp prevent accurate acquisition and assembly of complete transcripts, thus hindering the identification of isoforms, homologous genes, superfamily genes, and allele genes, especially in cases with low-quality or non-existent reference genomes [41]. Single-molecule sequencing technology eliminates the need to disrupt RNA molecules, enabling precise determination of the entire structure of a single transcript and the subsequent derivation of a complete cDNA sequence [42,43]. Enhancing the ability to uncover intricate details can offer a deeper understanding of transcriptome complexity. Our study utilized RNA-Seq and full-length transcriptome data to pinpoint genes involved in metabolic herbicide resistance mechanisms.

P450s are the most prevalent enzymes involved in herbicide detoxification. However, owing to their vast quantity and complex functionality, fully exploring the potential of herbicide metabolism mediated by P450 remains challenging [44]. We identified three P450 genes (*CYP71C4*, *CYP90D2*, and *CYP72A15*) in *B. japonicus* that are important for metabolic resistance to mesosulfuron-methyl groups. These findings are similar to those of many other weeds with metabolic herbicide resistance, and approximately 30 P450s have been demonstrated to possess herbicide-metabolizing function [45]. For example, *CYP71D10* is significantly transcribed in *A. aequalis* with mesosulfuron-methyl resistance [25]. Several P450 genes (*CYP710A1*, *CYP74A*, *CYP96A13*, *CYP96B116*, *CYP76M51*, *CYP94A1*, *CYP77A3*, *CYP86A8*, and *CYP86A2*) are overexpressed in herbicide-resistant *M. aquaticum* [26], *D. sophia* [24], *P. fugax* [40], *A. aequalis* [25], and *C. bursa-pastoris* [28]. Several P450 genes that confer herbicide resistance have been identified in crop species, such as *CYP71A10* in soybean [46], *CYP749A16* in cotton [47], and *CYP71C6v1* in wheat [48]. Transgenic technology can enhance our understanding of the crucial role of P450s in herbicide resistance. For example, the *CYP76* family enhances the resistance of transgenic *Arabidopsis thaliana* to monoterpenol and phenylurea herbicides [49].

GST and GT are two additional enzyme families that play crucial roles in herbicide detoxification. These enzymes can either directly bind to herbicides or catalyze the reactions involved in their metabolism [6]. We identified two GST genes (*MEE6.28* and *GSTZ5*) and one GT gene (*SGT31*) in *B. japonicus* exhibiting metabolic resistance. Previous studies reported that GST-mediated metabolic resistance is an important factor in the high resistance of *Amaranthus palmeri* to atrazine [50]. Tau-, Phi-, Cys-, and Ser-GSTs are primarily responsible for herbicide detoxification in plants [51,52]. Dehydroascorbate reductase (DHAR), a GST member, is important for GSH binding and oxidized glutathione release [53], and is more active in wheat than in *L. multiflorum* Lam [54]. Similarly, ALS-inhibiting herbicides elicit an increase in GT gene activity in *A. thaliana* [55]. *UGT90A1* and *UGT83A1* in *B. syzigachne* [56] and *GST23* and *UGT73C5* in *P. fugax* [40] were overexpressed, leading to metabolic resistance. These findings imply that the overexpression of GST and GT in the *B. japonicus* population could potentially contribute to mesosulfuron-methyl resistance. Moreover, metabolic detoxification observed in *A. myosuroides* is partially attributed to the elevated expression of P450s and GSTs, ultimately resulting in enhanced O-glucosyl transferase activity [57]. This implies that the herbicide defense mechanism operates in collaboration with multiple gene families, necessitating further investigation to elucidate the precise roles of P450s, GSTs, and GTs.

Compared with P450s, GSTs, and GTs, ABC transporters generally segregate herbicides and their metabolites to express resistance [6]. In the present study, two ABC transporters (*ABCC10* and *ABCC2*) were found to be involved in *B. japonicus* resistance, supporting the results of previous studies on herbicide detoxification [58]. Similarly, *AB8G* and *AB14G* are related to the resistance of *C. bursa-pastoris* to tribenuron-methyl [28]. *ABCC* and *ABCG* are likely to participate in fenoxaprop-p-ethyl metabolic resistance in *P. fugax* [40]. Moreover, the ABC transporter *AtOPT6* may confer resistance to primisulfuron in *A. thaliana* by transporting glutathione derivatives [59]. Overexpression of *ABCC8* can also confer resistance to glyphosate in several crops, such as rice, soybean, and maize [60]. Therefore, the ABC transporter is likely involved in mesosulfuron-methyl resistance in *B. japonicus*, indicating that this population may have two NTSR mechanisms: an enhanced metabolism and a protective mechanism. This protective mechanism warrants further investigation in the future.

We identified two other genes encoding hydrolases and oxidases that are likely associated with mesosulfuron-methyl metabolic resistance in *B. japonicus*. Hydrolases can cleave herbicide molecules and, subsequently, oxidases transform them into hydrophilic metabolites [6]. This process shields plant cells from oxidative damage triggered by herbicides, thereby contributing to herbicide resistance [3].

NTSR serves as a universally adaptive trait for weeds, potentially granting resistance to a diverse array of herbicides [61]. We speculated that the NTSR mechanism exhibited by the TJ07 population may confer broad-spectrum resistance to other herbicides. To gain insight into the potential metabolic resistance mechanisms of *B. japonicus*, further experiments are needed to verify the resistance functions and mechanisms of the candidate genes in subsequent stages.

## 4. Materials and Methods

### 4.1. Plant Materials

Seeds of the resistant *B. japonicus* population (TJ07) were collected from wheat fields in Tianjin China, in 2018, where mesosulfuron-methyl failed to control this weed. The seeds, harvested from a minimum of 50 mature plants randomly scattered across the field, were thoroughly blended, air-dried, and subsequently stored in paper bags at a temperature of 4 °C for future utilization. The susceptible population (TJ01) (hereafter designated as S) was described by Li et al. [33]. Prior to planting, seeds were sown on 9 cm Petri dishes lined with two layers of Whatman No. 1 filter paper (Maidstone, UK) moistened with 5 mL distilled water. Petri dishes were kept in growth chambers at 25 °C and for a 12 h photoperiod (Model RXZ, Ningbojiangnan Instrument Factory, Ningbo, China). After four days, 90 germinated seeds were transplanted into six plastic pots (11 cm × 9 cm) filled with mixed soil (50% organic matter and moist loam soil, pH 5.6). The pots were randomly placed in a controlled greenhouse, and watered every 48 h. *B. japonicus* seedlings were thinned to ten seedlings that were evenly sized at the two- to three-leaf stage, and treated with herbicides when they reached the three- to four-leaf stage. Herbicides were applied using a laboratory sprayer equipped with a flat fan nozzle delivering 450 L ha^−1^ at 275 kPa. After spraying, the weeds were returned to the greenhouse, and the surviving seedlings (plants with new growth were considered alive) or biomass (aboveground dry weight) were recorded 21 d after treatment (DAT). Finally, there were 40 surviving plants.

Forty plants that survived treatment with 27 g a.i. ha^−1^ (2-fold higher than the field-recommended rate) of mesosulfuron-methyl (30 g L^−1^ OD; Bayer, Hangzhou, China) were selected for ALS gene sequencing. Twenty resistant plants with no known ALS resistance mutations were isolated before flowering, and seeds were obtained and used for a second round of mesosulfuron-methyl resistance selection (27 g a.i. ha^−1^). Plants surviving the second round of selection were also isolated to produce seeds (hereafter designated as MR), which were used for subsequent experiments.

### 4.2. Effect of Malathion on Mesosulfuron-methyl Resistance

Whole-plant dose–response experiments were conducted to determine the GR_50_ values of the MR and S populations for mesosulfuron-methyl in the absence and presence of malathion (45% EC; Huayu, Tianjin, China). Malathion was applied at a rate of 1000 g a.i. ha^−1^, with no negative effects on *B. japonicus* seedling growth [25,28]. Mesosulfuron-methyl was sprayed 1 h after malathion application at rates of 0, 3, 9, 27, 81, 243, and 729 g a.i. ha^−1^ for MR plants and 0, 0.11, 0.33, 1, 3, 9, and 27 g a.i. ha^−1^ for S plants. Herbicides were applied using a laboratory sprayer equipped with a flat fan nozzle delivering 450 L ha^−1^ at 275 kPa. After spraying, weeds were transferred to a greenhouse. Aboveground shoots were harvested 21 DAT, dried at 80 °C for 72 h, and the dry weights were recorded. Each treatment had three replicates, and the experiment was performed twice.

Data from the repeated experiments were analyzed using ANOVA with SPSS software (version 22.0; IBM Corporation, Armonk, NY, USA), and there was no significant (*p* < 0.05) trial-by-treatment interaction between the repeated experiments. Data were pooled and fitted to a nonlinear regression analysis using SigmaPlot (version 13.0; Systat Software, Inc., San Jose, CA, USA). The herbicide dose causing 50% growth reduction (GR_50_) was evaluated using a four-parameter log-logistic equation as follows:

y = c + (d − c)/(1 + (x/GR_50_)^b^)

where c is the lower limit, d is the upper limit, b is the slope of GR_50_, x is the independent variable (herbicide dose), and y is the dependent variable (percentage of dry weight). The resistance index (RI) was calculated as the ratio of MR GR_50_ to S GR_50_.

### 4.3. ALS Gene Sequencing

Genomic DNA was extracted from young leaf tissues of each *B. japonicus* plant at the three- to four-leaf stage using an EasyPure Plant Genomic DNA Kit (TranGen Biotech, Beijing, China). The primers used to amplify the *B. japonicus* ALS gene fragment and the PCR protocol have been described by Li et al. [33]. The amplification products were purified using an EasyPure Quick Gel Extraction Kit (TransGen Biotech, Beijing, China) and sequenced by GENEWIZ Biotech Co., Ltd. (Beijing, China). The ALS gene was aligned and compared using DNAMAN v.6.0.3 software (Lynnon Biosoft, Quebec, QC, Canada).

### 4.4. UPLC-MS/MS Analysis of Mesosulfuron-methyl Residue in B. japonicus Plants

Mesosulfuron-methyl (4 μg per plant) was applied to *B. japonicus* plants of MR and S populations at the three- to four-leaf stage using micropipettes. Ten plants were randomly collected from the MR and S *B. japonicus* populations at 1, 3, 5, 7, and 9 DAT. These plants were thoroughly washed with 20 mL acetonitrile, and subsequently frozen in liquid nitrogen for storage at −20 °C. Mesosulfuron-methyl extraction and residue analysis were performed as previously reported [26]. Validation of the analytical methods is presented in Table S1 and Figure S5. The absorption rate was calculated by dividing the amount of absorbed mesosulfuron-methyl by the applied amount of mesosulfuron-methyl, multiplied by 100%, while the metabolism rate was determined by dividing the metabolized amount of mesosulfuron-methyl by the absorbed amount, also multiplied by 100%. Each treatment had three replicates, and the experiment was performed twice.

### 4.5. Whole-Transcriptome Sequencing

Leaf samples were collected from herbicide-treated and untreated plants 24 h after the application of mesosulfuron-methyl (T; 9 g a.i. ha^−1^). Each treatment had three biological replicates, and 12 samples were collected (three biological replicates × two treatments × two populations). All samples were frozen immediately in liquid nitrogen and stored at −80 °C until RNA extraction.

Total RNA was extracted using Transzol Up (TransGen Biotech, Beijing, China) according to the manufacturer’s protocol and treated with DNase I (Takara, Beijing, China). RNA quality was monitored using 1% agarose gels, a Qubit 3.0 Fluorometer (Life Technologies, Carlsbad, CA, USA), and an RNA Nano 6000 Assay Kit of the Bioanalyzer 2100 system (Agilent Technologies, Santa Clara, CA, USA). High-quality RNA was used for cDNA library construction, Illumina sequencing, and isoform sequencing (Iso-Seq) by Biomarket Technologies Co., Ltd. (Beijing, China) using the NovaSeq 6000 platform (Illumina, San Diego, CA, USA).

Oligo (dT)-enriched RNA with a PolyA tail was reverse-transcribed into cDNA using a SMARTer PCR cDNA Synthesis Kit. The cDNA was then amplified and purified using 0.8X AMPure PB beads (PacBio, Menlo Park, CA, USA) for the construction of PacBio full-length Iso-Seq libraries. After screening the fragments, an SMRTbell Template Prep kit (version 2.0) was used to construct the SMRTbell library for damage repair and end repair. After the library was quantified, a PacBio Binding Kit was used to combine the library template and enzyme complex, which were then transferred to the nanopores of a PacBio Sequel II sequencer for computer sequencing. The transcripts of the full-length sequences were used to predict open reading frames (ORFs) and long noncoding RNAs (lncRNAs), and the transcriptome data of the second generation were used for transcriptome quantitative, differential, and functional enrichment analyses.

To refine protein-coding sequence (CDS) prediction for redundant transcripts, we utilized TransDecoder (version 3.0.1). Among the transcripts, six open reading frames (ORFs) exceeding 300 bp in length and possessing a positive log-likelihood score for their approximate function were evaluated. The ORF with the highest score among those retained in the CDS was chosen, and specifically, the longest ORF transcripts were selected for further redundancy analysis. Gene function was annotated using eight publicly accessible databases: NCBI nonredundant protein sequences (NR), a manually annotated and reviewed protein sequence database (Swiss-Prot), Cluster of Orthologous Groups (COG), euKaryotic Ortholog Groups (KOG), Protein Family (PFAM), Gene Ontology (GO), Kyoto Encyclopedia of Genes and Genomes (KEGG), and evolutionary genealogy of genes: Non-supervised Orthologous Groups (eggNOG). Next, GO annotation and functional classification were implemented using Blast2GO and WeGO software, respectively. Pathways were assigned using the KEGG Automatic Annotation Server (http://www.genome.jp/kegg/kaas/) (accessed on 9 October 2023).

### 4.6. Identification and Analysis of Differentially Expressed Genes (DEGs)

Full-length transcripts served as the reference to identify differentially expressed transcripts between the comparison groups. Second-generation sequencing data were used for comparison and quantification, and the quantitative read count was used to compare the differences between groups. Differential expression abundance for each gene per transcript between sample pairs was calculated using DESeq2 v.1.4.5. Genes with a |log2(foldchange)| ≥ 1 and a false discovery rate (FDR) < 0.01 were identified as differentially expressed genes (DEGs). Comparisons of expression differences were conducted between untreated MR and untreated S samples at time 0 (MR_CK and S_CK), and between MR and S samples at 24 h post mesosulfuron-methyl treatment (MR_T and S_T). Further comparisons were made between MR and S samples for both untreated (CK) and treated (T) conditions. DEGs were further analyzed through Gene Ontology (GO) and Kyoto Encyclopedia of Genes and Genomes (KEGG) enrichment analyses using a hypergeometric test, with GO or KEGG terms considered significantly enriched if q < 0.05.

### 4.7. Selection and Validation of Candidate Metabolic Resistance Genes

We selected 15 overlapping genes identified as mesosulfuron-methyl metabolism-related genes using transcriptome annotation that were significantly upregulated in MR_T relative to S_T and in MR_T relative to MR_CK. Glyceraldehyde-3-phosphatedehydrogenase genes (GAPDH, JN599100.1) were chosen as an internal control to measure gene expression and as internal control genes to ensure the reliability of qRT-PCR [62]. Gene-specific primers used for qRT-PCR are listed in Supplementary Table S2.

qRT-PCR was performed on an ABI StepOne Plus real-time PCR system (ABI, Carlsbad, CA, USA) using SuperReal PreMix Plus (SYBR Green) (Tiangen, Beijing, China). The 20 µL mixture reaction contained 10 µL of SYBR Mix, 0.6 µL of forward primer, 0.6 µL of reverse primer, 1 µL of cDNA, 0.4 µL of ROX Reference Dye, and 7.4 µL of RNase-free ddH2O, and there were three replicates per cDNA sample. The qRT-PCR programs were as follows: 95 °C for 15 min, followed by 40 cycles of 95 °C for 10 s and 60 °C for 30 s. To ensure primer specificity, melting curve analysis was conducted at the reaction’s end, with a similar amplification efficiency observed for both target and internal control genes. Fold change in gene expression was calculated using the comparative cycle threshold (CT) method, expressed as 2^−ΔCT^.

Statistical analysis of the data was conducted using Student’s *t*-test (*p* < 0.05) in SPSS software (SPSS Inc., Chicago, IL, USA).

The expression patterns of these genes were measured using qRT-PCR using the original RNA samples used for RNA-Seq. Then they were also examined in the other plants of MR and S populations as parallel materials collected at 72 h after mesosulfuron-methyl treatment [26,63].

## 5. Conclusions

In summary, our study demonstrated that *B. japonicus* population TJ07 has P450-involved metabolic resistance and ABC transporter-involved protective resistance to mesosulfuron-methyl. RNA-Seq and full-length transcriptome analyses provided abundant gene information on herbicide metabolism and revealed the genes related to mesosulfuron-methyl metabolism in a *B. japonicus* subpopulation with two generations of herbicide selection. Metabolic resistance in TJ07 is likely to be comprehensively regulated by multiple genes, including P450s, GSTs, GTs, and ABC transporters. Future studies should aim to elucidate these relationships.

## Figures and Tables

**Table 1:** GR_50_ values of susceptible (S) and resistant (MR) *B. japonicus* populations’ response to mesosulfuron-methyl with or without malathion pre-treatment.

Herbicide	GR_50_ ± SE (g a.i. ha^−1^) ^1^		RI ^2^
	S	MR	
Mesosulfuron-methyl	0.90 ± 0.17 a ^3^	46.64 ± 3.63 a	51.8
Mesosulfuron-methyl + Malathion	0.59 ± 0.21 a	7.83 ± 1.40 b	13.3

^1^ GR_50_, herbicide dose causing 50% growth reduction in *B. japonicus*; SE, standard error: *n* = 6. ^2^ Resistance index (RI) = GR_50_(MR)/GR_50_(S). ^3^ Means with different letters in a row for each herbicide are significantly different by the Tukey’s HSD test (α = 0.05).

**Table 2:** Sequence alignment and deduced amino acid of the resistant and susceptible *B. japonicus* populations.

Population	The Amino Acid Position, Relative Nucleotide, and Amino Acid							
	122	197	205	376	377	574	653	654
TJ01	GCA	CCT	GCG	GAT	CGT	TGG	AGT	GGT
	Ala	Pro	Ala	Asp	Arg	Trp	Ser	Gly
TJ07	GCA	CCT	GCG	GAT	CGT	TGG	AGT	GGT
	Ala	Pro	Ala	Asp	Arg	Trp	Ser	Gly

Note: the amino acid sequence is based on the ALS sequence of *Arabidopsis thaliana* (AY042819.1); the mutated amino acid and corresponding codon are underlined.

**Table 3:** Sequence annotation of the *B. japonicus* transcriptome.

Public Database	Number of Transcripts	Percentage (%)
Annotated in NR	33,076	99.56
Annotated in Swiss-Prot	22,712	68.37
Annotated in KEGG	14,199	42.74
Annotated in KOG	19,476	58.63
Annotated in eggNOG	31,856	95.89
Annotated in PFAM	24,835	74.76
Annotated in GO	27,312	82.21
Annotated in COG	13,728	41.32

**Table 4:** The fifteen enriched KEGG pathway terms of the DEGs between mesosulfuron-methyl-treated MR and S *B. japonicus* populations.

KEGG Pathway Term	Map ID	Gene Count ^1^		Genes in Background ^2^	*p*-Value
		Up	Down		
Starch and sucrose metabolism	map00500	81	75	358	2.85 × 10^−3^
Phenylpropanoid biosynthesis	map00940	51	53	203	1.00 × 10^−5^
Cyanoamino acid metabolism	map00460	35	43	133	1.37 × 10^−7^
Glutathione metabolism	map00480	34	65	209	7.09 × 10^−4^
Cysteine and methionine metabolism	map00270	34	56	205	1.62 × 10^−2^
Ribosome biogenesis in eukaryotes	map03008	31	59	194	2.65 × 10^−3^
Fatty acid degradation	map00071	28	20	100	1.16 × 10^−2^
Aminoacyl-tRNA biosynthesis	map00970	27	35	118	2.45 × 10^−4^
Photosynthesis—antenna proteins	map00196	24	45	140	1.20 × 10^−3^
alpha-Linolenic acid metabolism	map00592	23	27	102	6.12 × 10^−3^
Sulfur metabolism	map00920	23	28	105	7.11 × 10^−3^
Ubiquinone and other terpenoid-quinone biosynthesis	map00130	21	21	82	4.33 × 10^−3^
beta-Alanine metabolism	map00410	17	24	83	1.05 × 10^−2^
Isoquinoline alkaloid biosynthesis	map00950	14	15	56	1.35 × 10^−2^
Monoterpenoid biosynthesis	map00902	5	7	15	7.35 × 10^−4^

^1^ Number of up- and downregulated genes enriched in this pathway. ^2^ Number of genes annotated in this pathway.

**Table 5:** Identification of upregulated genes related to metabolic-resistance in *B. japonicus* by RNA-Seq and qRT-PCR (2^−ΔCt^) ^1^.

Gene ID	PFAM ID	Function Annotation	RNA-Seq		qRT-PCR (2^−ΔCt^)	
			log2 Fold Change (MR_T vs. S_T)	Padj	RNA-Seq Samples (MR_T vs. S_T)	Additional Samples (MR_T vs. S_T)
E_transcript_38404	PF00067.21	CytP450, CYP71C4	2.18	7.30 × 10^−15^	1.01	2.28
E_transcript_8113	PF00067.21	CytP450, CYP71C4	2.41	1.56 × 10^−26^	2.86 *	6.60 *
E_transcript_31473	PF00067.21	CytP450, CYP90B1	1.29	5.11 × 10^−6^	1.03	58.42
E_transcript_5503	PF00067.21	CytP450, CYP71C1	2.25	1.03 × 10^−21^	0.71	43.16
E_transcript_6685	PF00067.21	CytP450, CYP71C2	1.89	5.02 × 10^−16^	0.60 *	20.76
E_transcript_13219	PF00067.21	CytP450, CYP71C2	2.16	1.10 × 10^−19^	0.65 *	3.05 *
E_transcript_5754	PF00067.21	CytP450, CYP90D2	1.02	1.51 × 10^−4^	1.29 *	9.37 *
E_transcript_48605	PF00067.21	CytP450, CYP71C	2.73	5.75 × 10^−20^	0.76 *	8.11
E_transcript_71401	PF00067.21	CytP450, CYP72A15	2.27	2.22 × 10^−23^	7.13 *	14.27 *
E_transcript_44243	PF02798.19	GST, MEE6.28	1.25	4.63 × 10^−7^	1.71 *	9.12 *
E_transcript_14304	PF13410.5	GST, GSTZ5	8.12	1.72 × 10^−150^	3.31 *	18.27 *
E_transcript_38926	PF02798.19	GST, PUR7	1.43	5.46 × 10^−7^	1.77 *	37.08
E_transcript_56910	PF00201.17	UDP-glucosyl transferase, SGT31	2.84	2.26 × 10^−17^	5.17 *	5.50 *
E_transcript_53473	PF00005.26	ABC transporter, ABCC10	1.52	6.64 × 10^−6^	1.62 *	20.18 *
E_transcript_71047	PF00005.26	ABC transporter, ABCC2	6.89	5.78 × 10^−118^	2.72 *	1.47 *
E_transcript_5168	PF03109.15	ABC1 family, At5g05200	1.20	2.21 × 10^−4^	1.13	3.24
E_transcript_15373	PF01786.16	Oxidase, MCB17.11	1.30	2.48 × 10^−6^	2.53	2.66
E_transcript_48732	PF00724.19	Oxidase, OPR11	1.73	1.31 × 10^−11^	1.21 *	1.58 *
E_transcript_65166	PF12697.6	Hydrolase	1.62	2.54 × 10^−6^	3.34	2.68
E_transcript_19506	PF01738.17	Hydrolase	1.20	6.84 × 10^−3^	1.26	3.42
E_transcript_38621	PF01156.18	Hydrolase	1.61	2.09 × 10^−4^	2.23 *	4.83 *

^1^ MR_T, resistant *B. japonicus* plants treated with mesosulfuron-methyl. S_T, susceptible *B. japonicus* plants treated with mesosulfuron-methyl. The obtained *p*-value was adjusted and represented as the q-value through application of the Benjamini–Hochberg procedure, aiming to effectively manage the false discovery rate. * Significant differences between MR_T and S_T at a 0.05 level according to Fisher’s protected LSD test.

## Data Availability

Data are available from the author.

## Supplementary Materials

The following supporting information can be downloaded at https://www.mdpi.com/article/10.3390/plants13131751/s1: Figure S1: Species distributions of the BLASTX matches of *B. japonicus* transcriptome genes; Figure S2: GO function classification of the annotated genes in *B. japonicus*. The genes were allocated to three categories: cellular component, molecular function, and biological process; Figure S3: KEGG function classification results of the annotated genes in *B. japonicus*. The y-axis lists the various KEGG pathways. The x-axis indicates the number of genes. According to their participation in these KEGG pathways; the genes were divided into five color-coded groups: metabolism, genetic information processing, cellular processes, environmental information processing, and organismal systems; Figure S4: The annotated DEGs between the MR_T vs. S_T samples using GO (A) and KEGG (B) enrichment analyses; Figure S5: Typical chromatograms of mesosulfuron-methyl from extracted *B. japonicus* samples; Table S1: The analysis method of mesosulfuron-methyl in *B. japonicus* using QuEChERS and UPLC-MS/MS was validated in terms of its linearity, limit of quantification (LOQ), accuracy, and precision; Table S2: Primer pairs used for qRT-PCR relative quantification of gene expression in *B. japonicus.*
