# Supplementary material for: Metabolism-Based Herbicide Resistance to Mesosulfuron-methyl and Identification of Candidate Genes in Bromus japonicus
Source: Plants (Basel). 2024 Jun 25;13(13):1751. doi: 10.3390/plants13131751 (PMC11244151; doi:10.3390/plants13131751)
Supplement: Supplementary file 1 [file plants-13-01751-s001.zip › plants-3058582-supplementary.pdf]

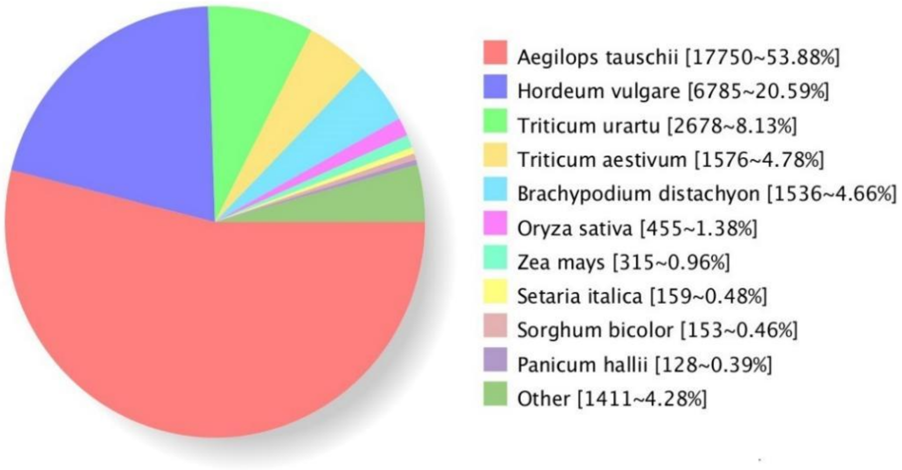

Figure S1. Species distributions of the BLASTX matches of the *B. japonicus* transcriptome genes.

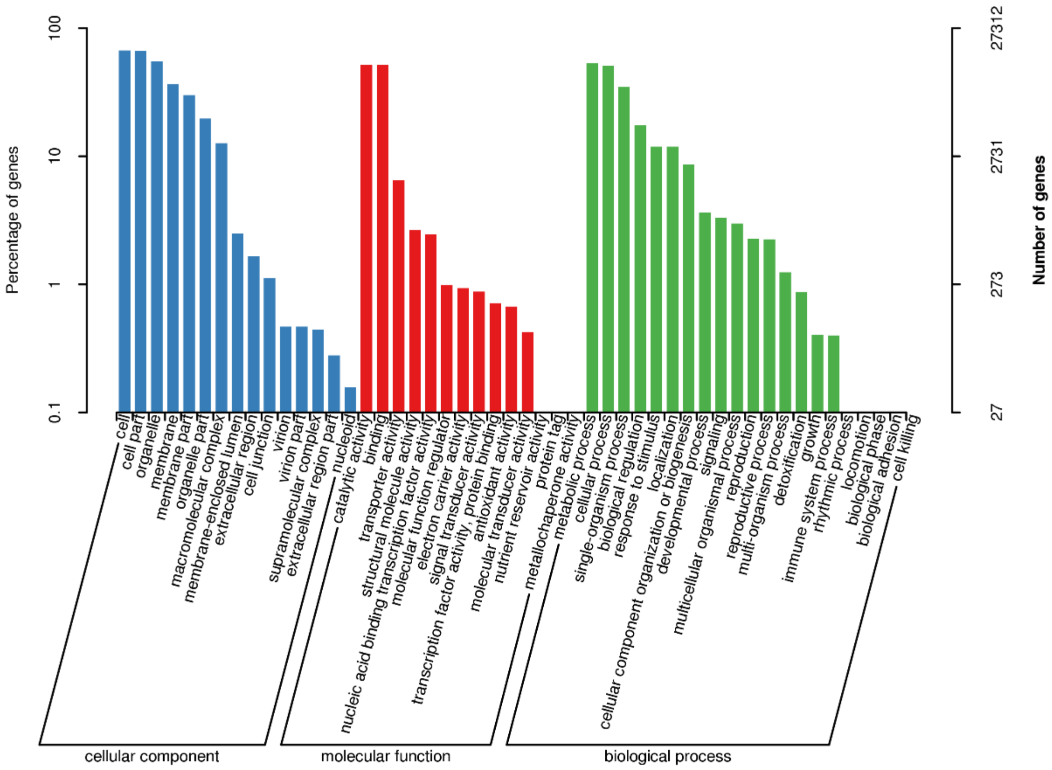

Figure S2. GO function classification of the annotated genes in *B. japonicus*. The genes were allocated to three categories: cellular component, molecular function, and biological process.

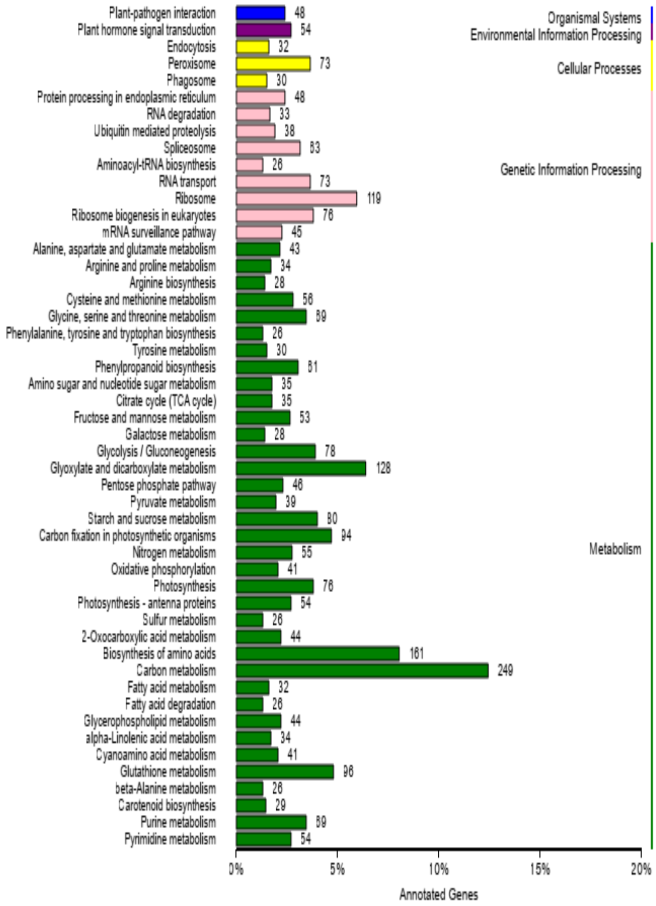

Figure S3. KEGG function classification results of the annotated genes in *B. japonicus*. The y-axis lists the various KEGG pathways. The x-axis indicates the number of genes. According to their participation in these KEGG pathways, the genes were divided into five color-coded groups: metabolism, genetic information processing, cellular processes, environmental information processing, and organismal systems.

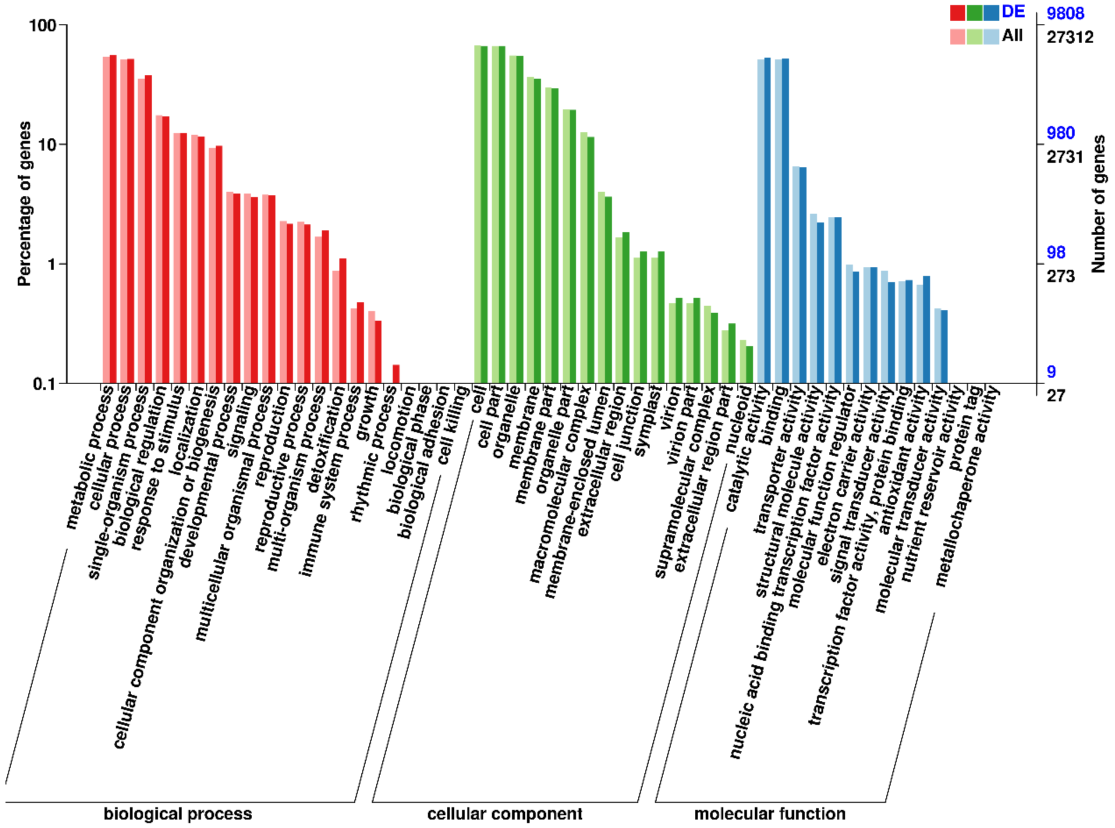

DE
All
Percentage of genes
100
10
1
0.1
Number of genes
9808
27312
980
2731
98
273
9
27
metabolic process
cellular process
single-organism process
biological regulation
response to stimulus
localization
cellular component organization or biogenesis
developmental process
multicellular organismal process
signaling
reproduction
reproductive process
multi-organism process
detoxification
immune system process
growth
rhythmic process
locomotion
biological phase
biological adhesion
cell killing
cell
cell part
organelle
membrane
membrane part
organelle part
macromolecular complex
membrane-enclosed lumen
extracellular region
cell junction
symplast
virion
virion part
supramolecular complex
extracellular region part
nucleoid
catalytic activity
binding
transporter activity
structural molecule activity
nucleic acid binding transcription factor activity
molecular function regulator
electron carrier activity
signal transducer activity
transcription factor activity, protein binding
antioxidant activity
molecular transducer activity
nutrient reservoir activity
protein tag
metallochaperone activity
biological process
cellular component
molecular function

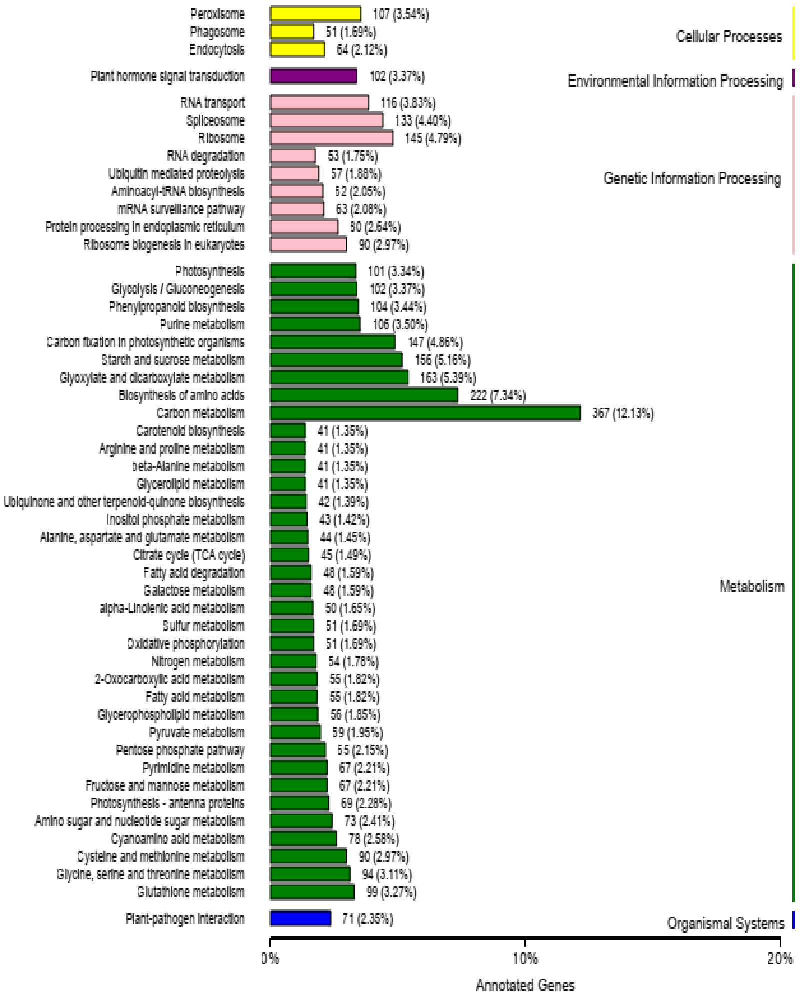

Figure S4. The annotated DEGs between the MR_T vs. S_T samples using GO *(A)* and KEGG *(B)* enrichment analyses.

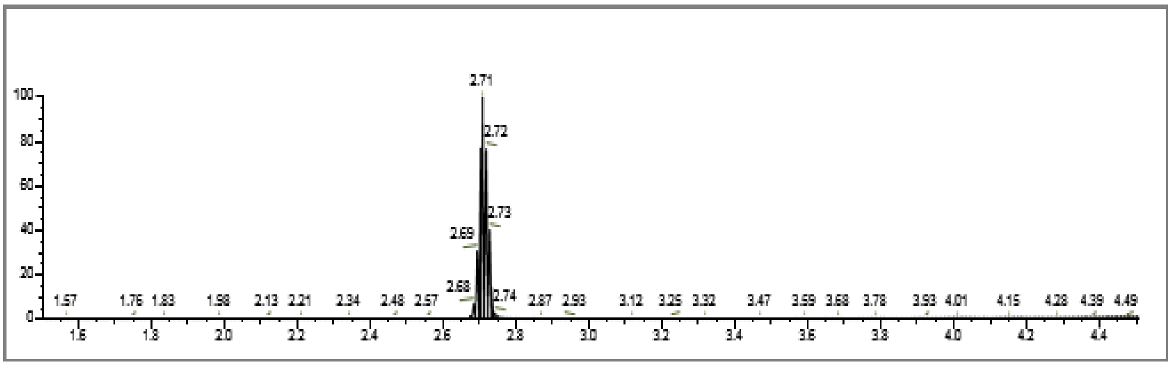

Figure S5. Typical chromatograms of mesosulfuron-methyl from extracted *B. japonicus* samples.

Table S1. The analysis method of mesosulfuron-methyl in *B. japonicus* using QuEChERS and UPLC-MS/MS was validated in terms of its linearity, limit of quantification (LOQ), accuracy and precision.

Samples	Calibration curve	R^2	LOQ[a] (mg kg^{-1})	Fortified concentration (mg kg^{-1})	Recoveries[b] (%)
Japanese brome	Y=4.421000x+8141	0.9993	0.005	0.005	100.2±14.9
				0.05	97.8±8.8
				0.1	98.8±2.1

[a] The LOQ was considered the lowest fortified concentration that can be quantified with acceptable accuracy and precision.

[b] Average recoveries (n=5) ± relative standard deviations (RSDs) of tribenuron-methyl in matrix at three fortified concentrations were used to assess accuracy and precision.

Table S2. Primer pairs used for qRT-PCR relative quantification of gene expression in *B. japonicus.*

Gene ID	Gene annotation	Primers	Sequence （5′-3′）
E_transcript_38404	CytP450, CYP71C4	F	GATTTCCAGTTCCTGCCTTTTG
		R	CATCCTTCTCTTCCACTCCGTC
E_transcript_8113	CytP450, CYP71C4	F	AAGGGAAGGGATTTCGGGC
		R	AAGCAATAGACAAGGTTGGCGAG
E_transcript_31473	CytP450, CYP90B1	F	CACCAACACAGCCAGCGAGAG
		R	GGACGAAGGGGAGGAAGAAAAG
E_transcript_5503	CytP450, CYP71C1	F	CGACATCCTCTTCTACGGCTC
		R	AGTATTTGTTACCTCGTTCTCGC
E_transcript_6685	CytP450, CYP71C2	F	CTCCCAGTAGGCTCCCCGTC
		R	GGTGAAGGCGTTGAGCAGG
E_transcript_13219	CytP450, CYP71C2	F	GCGACCTGCTCAACGCCTTC
		R	TCGTCCCACATCTTGTTCACCTTC
E_transcript_5754	CytP450, CYP90D2	F	CTCGCCGTCAAGTTCCTCAG
		R	GCAAGGTTTCACCCAAGTCAG
E_transcript_48605	CytP450, CYP71C	F	AACGAGGTGAGGTTGGTGGTG
		R	GGTGAAGGCGTTGAGCAGGT
E_transcript_71401	CytP450, CYP72A15	F	AACAATCAAATGGGAAGGCAAAAC
		R	GGTGTGGTTCTTCCAAAGTGATGC
E_transcript_44243	GST, MEE6.28	F	CGAGGAGGTCACCGTGGATG
		R	CAAAGAGCCTGAATCTACCGTCG
E_transcript_14304	GST, GSTZ5	F	TGTCATCTCCGCCAAAGAAAATC
		R	CAACATACAACCTGGTCGTGCC
E_transcript_38926	GST, PUR7	F	ATCGCTGAGTCCACCATCATCGT
		R	GGTCGCCGTGAAGACAGGGTAG
E_transcript_56910	UDP-glucosyl	F	GTCGGAGCACAGCAAAAAGAAC

	transferase, SGT31	R	CTTGTCAACAGGGAATAGCACG
E_transcript_53473	ABC transporter, ABCC10	F	CTTCGTTTCTTCCACTTTCGTTG
		R	CCTATTGTTTGGTAATCCTCTTCATC
E_transcript_71047	ABC transporter, ABCC2	F	TGATTGCTGCTGTTTTCCGTAAG
		R	GACTCCGCATCGGTTGAAATC
E_transcript_5168	ABC1 family, At5g05200	F	CTCAGTTGCGGCTTGGTTTGC
		R	ATAAGGAACAGGTGGTGCTCGGTC
E_transcript_15373	Oxidase, MCB17.11	F	TCAAGTCCCTCCGCTTCCCCAC
		R	GAAGACCCCCTGGACGGTGATG
E_transcript_48732	Oxidase, OPR11	F	GTGAAAATGCCTTACCTAACCCAG
		R	TGAGACTGTAGCCAAACCTATGCC
E_transcript_65166	Hydrolase	F	TGAGCCAAGGTTCATCAATACTGTG
		R	GTTTCGGAAGAAGAAGCCCTGTG
E_transcript_19506	Hydrolase	F	GAAAACGCTGACAGACCAATACAAG
		R	GTAACAAAAGACGGGTGTGACATAAC
E_transcript_38621	Hydrolase	F	GATACCAATCACTGAGCCCTTCTTC
		R	CATTATTGAGAGTGCCACACCAGAC
